# Evaluation of genetic diversity and structure of the endangered medicinal plant genus *Huperzia* (Lycopodiaceae) in China based on EST–SSR markers

**DOI:** 10.3897/phytokeys.274.180449

**Published:** 2026-04-24

**Authors:** Muzi Li, Dengpan Yin, Haibo Li, Yumo Yue, Haibin Zhou, Wei Yu, Rui Qin, Zhongnian Zhang, Jiahao Wen, Yeqin Xu, Chaokai Miao, Qinyuan Shen, Dekai Wang

**Affiliations:** 1 Key Laboratory of Plant Secondary Metabolism Regulation in Zhejiang Province, College of Life Sciences and Medicine, Zhejiang Sci-Tech University, Hangzhou 310018, Zhejiang, China College of Life Sciences and Medicine, Zhejiang Sci-Tech University Hangzhou China; 2 Yuyao City Seed and Seedling Management Station, Ningbo 315499, Zhejiang, China Yuyao City Seed and Seedling Management Station Ningbo China; 3 Ningbo Liwah Pharmaceutical Co., Ltd, Ningbo 315174, China Ningbo Liwah Pharmaceutical Co., Ltd Ningbo China

**Keywords:** Genetic diversity, *Huperzia serrata*, SSR markers, transcriptome sequencing

## Abstract

*Huperzia serrata* is an endangered perennial medicinal lycophyte, primarily distributed across most regions of China, eastern and southern Asia, Oceania, and the Americas. It produces Huperzine A (HupA), a potent natural acetylcholinesterase (AChE) inhibitor with significant therapeutic potential for treating Alzheimer’s disease. To elucidate the genetic diversity and phylogenetic relationships of this species and facilitate its conservation, we performed full-length transcriptome sequencing of *H. serrata* using the PacBio Sequel II platform and developed expressed sequence tag–simple sequence repeat (EST–SSR) markers. Transcriptome analysis identified 16,511 SSR loci from the transcriptome, with an average density of one locus per 4.11 kb. These loci exhibited diverse repeat motifs (ranging from mono- to hexanucleotides), predominantly containing 5–20 repeats. Genotyping of 47 *Huperzia* accessions (representing 8 different species) using 21 polymorphic primers identified 98 alleles, yielding mean Nei’s gene diversity (*H*) and Shannon’s information index (*I*) values of 0.3171 and 0.4819, respectively, indicating a moderate level of genetic diversity. Principal coordinate analysis (PCoA) revealed no significant correlation between genetic and geographic distance. Cluster analysis based on genetic similarity coefficients (ranging from 0.41 to 0.82) delineated distinct genetic groupings. However, SSR cluster analysis was inconsistent with taxonomic identification based on morphology to a great extent. At a similarity coefficient of 0.51, the accessions were classified into four major groups. Notably, accessions from Guizhou (GZ) and Yunnan (YN) provinces tended to cluster together, with accessions GS_1_GL and GZ_6_GY exhibiting the highest genetic similarity. This study provides the first comprehensive set of genomic resources and insights into the genetic diversity of *Huperzia* across a wide range, revealing its fine-scale genetic structure. These findings establish a crucial scientific foundation for the conservation and sustainable utilization of this valuable medicinal species.

## Introduction

The genus *Huperzia* Bernh., a member of the Lycopodiaceae family (PPG I 2016), encompasses around 55 species (Zhang and Iwatsuki 2013), predominantly thriving in tropical and subtropical zones. In China, a total of 25 species and one variety have been officially recorded, with the vast majority clustering in the southwestern and southern parts of the country, as well as the middle and lower reaches of the Yangtze River basin (Kung and Huang 2004). Qian Ceng Ta, a traditional Chinese medicine derived from the whole plant of *H. serrata* (Thunberg) Trevisan, was first documented in the Qing Dynasty text Zhi Wu Ming Shi Tu Kao. This herb has been traditionally used to treat various conditions, including lung abscesses, contusions, strains, swelling, poisoning, and schizophrenia (Cheng et al. 1986; Ma 2007).

The genus *Huperzia* produces various bioactive compounds, such as lycopodium alkaloids and triterpenoids. Among these, Huperzine A (HupA) is an effective, selective, and reversible acetylcholinesterase inhibitor known to enhance memory and aid in the treatment of Alzheimer’s disease (Ved et al. 1997; Ma et al. 2006; Ha et al. 2011). However, the limited genomic information and genetic data available for *Huperzia* have resulted in poor understanding of its structural characteristics, taxonomic classification, and interspecies relationships. Due to high morphological plasticity and the potential presence of cryptic species, traditional taxonomy based solely on morphology often falls short in accurately delimiting species boundaries within this genus (e.g., distinguishing *H. serrata* from the closely related *H. javanica* (Sw.) C.Y.Yang). This knowledge gap has significantly hindered comprehensive investigations into the taxonomic and structural specificity of the genus. Therefore, identifying hypervariable loci and developing molecular markers for species identification would greatly benefit both conservation efforts and phylogenetic studies of *Huperzia*.

Molecular markers are widely used for germplasm identification, population genetic structure analysis, and gene localization (Mahgoub et al. 2025; Teofanova et al. 2025). Simple sequence repeats (SSRs), also known as microsatellites, are tandemly repeated DNA motifs that can be genotyped using PCR-based molecular marker technology. They consist of tandemly repeated sequences of 1–6 nucleotide units, with each locus typically comprising dozens of nucleotides. These repeats are flanked by relatively conserved single-copy sequences and are widely distributed across the genome (Kalia et al. 2011).

Compared to other molecular markers, SSRs exhibit several distinct characteristics: (1) high abundance and relatively uniform genomic distribution, resulting in extensive polymorphism; (2) multiple alleles per locus, providing high informational content; and (3) co-dominant inheritance and good reproducibility, following Mendelian principles. Two common approaches are used for SSR marker development. The first is genomic SSR (gSSR), derived from non-coding regions of the genome. Although gSSRs carry substantial genetic information, their development process is often complex and costly (Eujayl et al. 2004). In contrast, expressed sequence tag-based SSRs (EST–SSRs) are developed from transcribed sequences within expressed gene regions. EST–SSRs can reflect gene expression variation, are relatively simple and cost-effective to develop, exhibit good transferability, and have been widely applied in plant genetic studies (Zeng et al. 2010). Owing to their considerable application potential, SSR markers have been extensively studied in many key crops and medicinal plants (Kashi et al. 2006; Wang et al. 2020). For example, in one study, 100 SSRs were randomly selected from the *Cunninghamia lanceolata* genome for primer design; 38 were successfully amplified, and 10 polymorphic SSRs were identified using 56 individuals (Han et al. 2013). Similarly, transcriptome analysis of *Pinellia ternata* revealed abundant SSR loci with high polymorphism potential (Wang et al. 2014). In another study, 29 polymorphic primer pairs developed from transcriptome data were used to distinguish eight *Camellia japonica* germplasms (Pan et al. 2019).

Therefore, in this study, we developed novel EST–SSR markers derived from the complete transcriptome of *H. serrata*. The genus *Huperzia* comprises approximately 55 species worldwide, with approximately 25 species distributed in China (Chu et al. 2025). By employing these diverse accessions to investigate genetic diversity, genetic structure, and phylogenetic relationships, we aim to provide a robust and highly transferable molecular toolkit. This approach not only establishes a scientific foundation for the conservation of *Huperzia* genetic resources, but also offers a reliable methodology for future systematic investigations, such as resolving complex taxonomic boundaries and comparing closely related species like *H. serrata* and *H. javanica*.

## Materials and methods

### Plant material and DNA extraction

Transcriptome sequencing was performed using fresh leaf samples of *H. serrata* collected from Yuyao City, Ningbo. The leaves were immediately frozen in liquid nitrogen and stored at −80 °C until use. Sequencing was conducted on the PacBio RSII platform by Nanjing Genepioneer Biotechnologies Inc. (Nanjing, China). The raw sequencing data have been deposited in the NGDC database under BioProject accession number PRJCA022941. For SSR marker screening and evaluation, a total of 47 *Huperzia* accessions collected from various regions (including Guizhou, Yunnan, Shaanxi, and Zhejiang) and at different periods were used (Suppl. material 1: table SS1). To rigorously evaluate the applicability of these markers across different taxa within the genus, we screened 47 *Huperzia* accessions encompassing 8 distinct species: *H. serrata* (11 accessions), *H. kunmingensis* Ching (6), *H. javanica* (6), *H. crassifolia* W. M. Chu & B. Y. Zhang ex Z. Y. Guo (6), *H. crispata* (Ching & H. S. Kung) Ching (6), *H. nanlingensis* Y. H. Yan & N. Shrestha (5), *H. selago* (L.) Bernh. ex Schrank & Mart. (4), and *H. sutchueniana* (Herter) Ching (3). These accessions were rigorously identified based on morphological characteristics (Flora of China, online) (https://www.iplant.cn) and collected from diverse regions, including Guizhou, Yunnan, Shaanxi, and Zhejiang. Young and healthy leaves from these accessions were selected, frozen in liquid nitrogen, and stored at −80 °C. Genomic DNA was extracted from all samples using a plant genomic DNA kit (Tiangen Biotech Co., Beijing, China).

### Identification of EST–SSR markers

SSR analysis was conducted on single genes screened greater than 1 kb using MISA software (Thiel et al. 2003). Parameters were established as follows: the number of single-to-hexanucleotide repeats was 10, 6, 5, 5, and 5 times, respectively (Merkel and Gemmell 2008; Ueno et al. 2012). The primers for the SSR sites were batch designed using Primer3 software (Untergasser et al. 2012). The parameters used in the primer design are as follows: annealing temperature (Tm) 57–63 °C, primer length 18–27 bp, Tm difference between the upstream and downstream primers 2 °C, expected length of the PCR product 100–300 bp, and avoidance of the appearance of secondary structure of the primer. A total of 100 pairs of SSR marker primers were randomly selected and synthesized by Sangon Biotech (Shanghai) Co., Ltd.

### Screening of polymorphic SSR primers and their application in species identification

An initial screening of the 100 synthesized SSR primer pairs was performed using two species, *H. crispata* and *H. serrata*, to identify polymorphic markers. The polymerase chain reaction (PCR) was carried out in a 20 µL mixture containing 1 µL of template DNA (30 ng/µL), 10 µL of 2× Taq PCR Master Mix (Novoprotein Scientific Co., Ltd.), 1 µL of each forward and reverse primer (10 µM), and 7 µL of ddH_2_O. The amplification protocol consisted of an initial denaturation at 94 °C for 5 min, followed by 35 cycles of denaturation at 94 °C for 30 s, annealing at 60 °C for 30 s, and extension at 72 °C for 1 min, with a final extension at 72 °C for 10 min before holding at 16 °C.

A volume of 5 µL of each PCR product was separated on an 8% non-denaturing polyacrylamide gel (PAGE) using 1× TBE as the running buffer. Electrophoresis was conducted at a constant voltage of 180 V for 120–140 min. Subsequently, the gel was stained with a solution prepared by mixing 60 µL of fluorescent dye (TOROIVD Tech. Co. Ltd.), 20 mL of 1 M NaCl, and 180 mL of ddH_2_O (total volume 200 mL). The gel was immersed in the staining solution and gently agitated for 30 min to ensure uniform staining. Finally, the gel was rinsed with ddH_2_O, and DNA bands were visualized and photographed under a UV transilluminator.

The SSR primers that exhibited clear polymorphism between *H. crispata* and *H. serrata* were selected for subsequent genotyping of all 47 *Huperzia* accessions.

### Data statistics and analysis

Amplification bands that were clear and reproducible across the PAGE assays were scored. The presence of a band was recorded as “1,” and its absence as “0,” to generate a binary data matrix. This matrix was compiled using Excel and served as the basis for all subsequent analyses. Genetic diversity parameters, including the mean number of alleles (Na) and Shannon’s information index (*I*), were calculated with POPGENE software (version 1.32) (Yeh et al. 1999). The polymorphism information content (PIC) for each primer was determined using PowerMarker (version 3.25) (Liu and Muse 2005).

To elucidate the genetic relationships among the 47 *Huperzia* accessions, a principal coordinate analysis (PCoA) was performed based on genetic distance matrices using GenALEx 6.5 (Peakall and Smouse 2006). Additionally, a cluster analysis was conducted using the unweighted pair group method with arithmetic mean (UPGMA) algorithm in NTSYS-pc (v2.0) (Rohlf 1992). The resulting genetic similarity dendrogram was visualized using the Tree Plot module within the same software package.

### Abbreviations

**HupA**, huperzine A; **EST–SSR**, expressed sequence tag–simple sequence repeats; **SSR**, simple sequence repeats; **PAGE**, polyacrylamide gel electrophoresis; **Na**, observed number of alleles; **PIC**, polymorphic information content; **H**, Nei’s genetic diversity index; **I**, Shannon diversity index; **PCoA**, principal coordinate analysis; **UPGMA**, unweighted pair group method with arithmetic mean.

## Results

### Repeat types and distribution of SSR loci in the transcriptome of *H. serrata*

**Figure 1.**
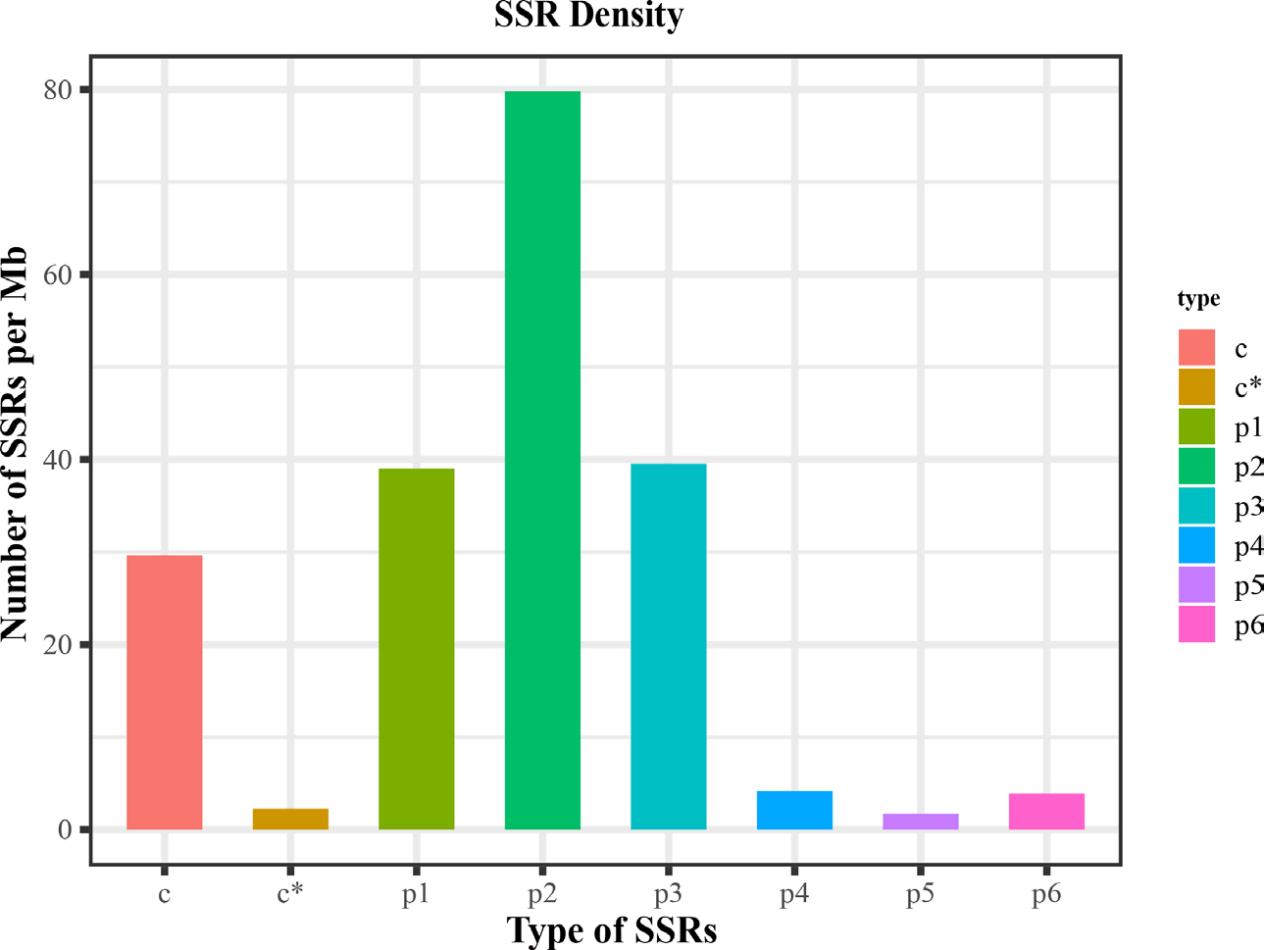
Distribution characteristics of nucleotide repeat types in different EST–SSR motifs of *H. serrata*. c, series does not embed complex repeat types; c*, series embedded complex repeat types; p1, mononucleotide; p2, dinucleotide; p3, trinucleotide; p4, tetranucleotide; p5, pentanucleotide; p6, hexanucleotide.

Full-length transcriptome sequencing of *H. serrata* was conducted using the PacBio SII platform, yielding 62.51 Gb of high-quality data. Subsequent assembly produced 31,243 unigenes with a total length of 67,873,943 bp and an average length of 2,172 bp. The assembly quality was robust, as indicated by an N50 value of 2,225 bp, with a GC content of 43.34%. We identified 16,511 SSR loci from 10,959 of the 31,243 unigenes, among which 2,934 were classified as compound SSRs. The SSR frequency was 35.08%, with one locus found every 4.11 kb on average. A total of 3,241 unigenes contained more than one SSR locus, demonstrating a rich abundance of SSRs in the *H. serrata* transcriptome. Analysis of the SSR repeat motifs revealed all types from mononucleotide to hexanucleotide repeats (Fig. 1). Dinucleotide repeats were the most abundant, with 8,341 loci accounting for 50.52% of all SSRs and an occurrence frequency of 26.70% across the unigenes. Mononucleotide and trinucleotide repeats were also common, whereas tetra-, penta-, and hexanucleotide repeats were comparatively rare (Table 1).

**Table 1.** Distribution characteristics of SSR repeat types in the transcriptome.

Repeat type	SSR number	Percentage (%)	Frequency (%)
mononucleotide (P1)	3,473	21.03	11.12
dinucleotide (P2)	8,341	50.52	26.70
trinucleotide (P3)	3,720	22.53	11.91
tetranucleotide (P4)	471	2.85	1.51
pentanucleotide (P5)	146	0.88	0.47
hexanucleotide (P6)	360	2.18	1.15
Total	16,511	100	52.86

### Characterization of SSR motif types in the transcriptome of *H. serrata*

**Figure 2.**
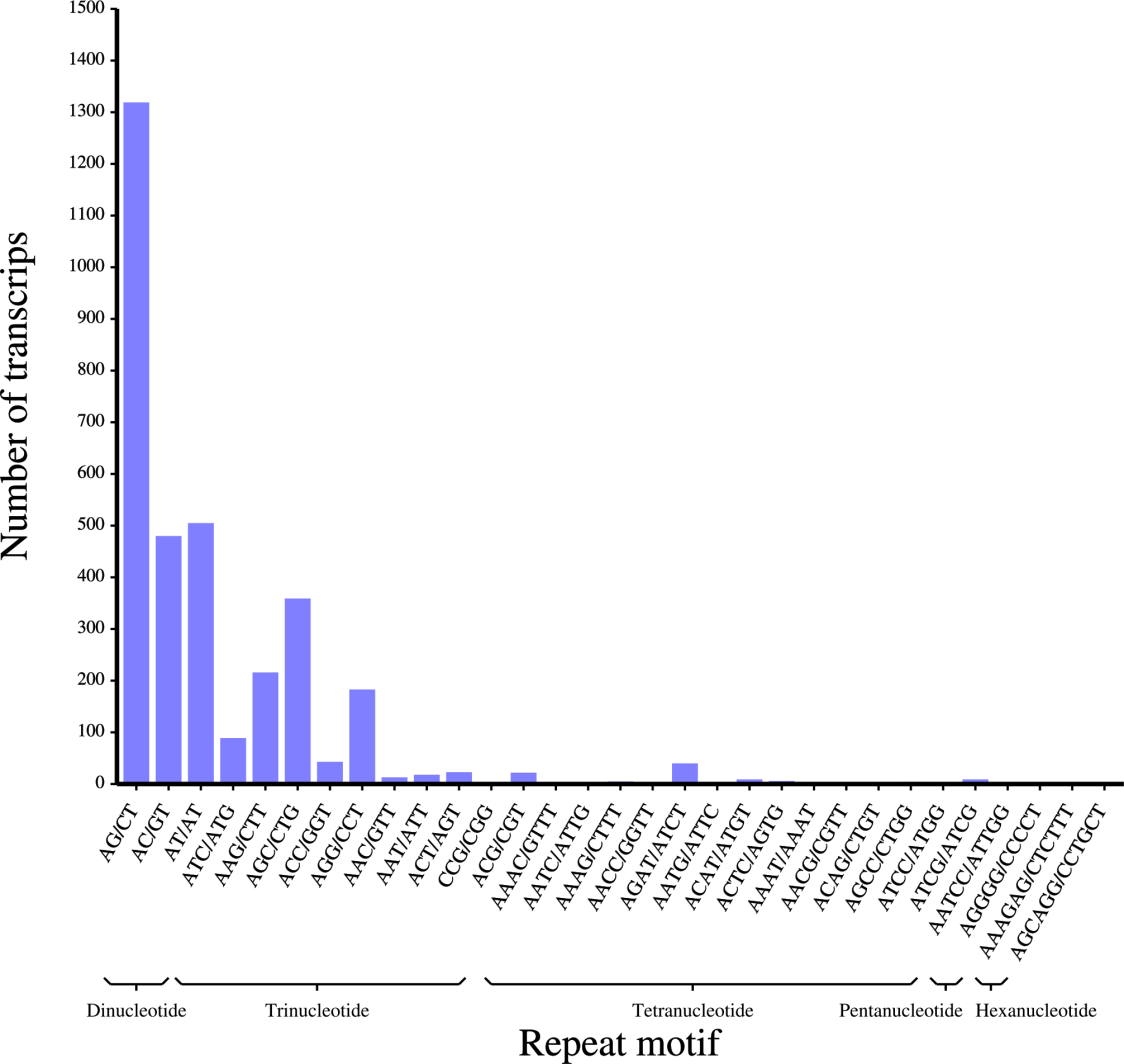
Distribution of repeat numbers among dominant SSR motifs of different repeat types.

In total, 141 distinct SSR repeat motif types were identified in the *H. serrata* transcriptome (Fig. 2; Table 2). Dinucleotide repeat motifs were the most prevalent, accounting for 45.25% of all SSR loci, followed by mononucleotide (16.30%) and trinucleotide (12.93%) motifs. Among the tetranucleotide repeats, the distribution of different motifs was relatively uniform, with the AGCG/CGCT motif exhibiting the highest proportion at merely 0.57%. For pentanucleotide and hexanucleotide repeats, the AGAGG/CCTCT and ATCGCC/ATGGCG motifs were the most abundant, with 33 and 72 occurrences, respectively. In contrast to the prevalent mono-, di-, and trinucleotide motifs, the tetra-, penta-, and hexanucleotide motifs were characterized by their lower overall occurrence, greater diversity of motif types, and a more dispersed distribution pattern across the transcriptome.

**Table 2.** Characteristics of repetitive motifs at SSR sites of the transcriptome.

Repeat type	Number of motif species	Repeating motif	Number	Percent (%)
mononucleotide (P1)	2	A/T	2695	16.30
		C/G	778	4.71
dinucleotide (P2)	4	AC/GT	1260	7.63
		AG/CT	6211	37.62
		AT/AT	787	4.77
		CG/CG	83	0.50
trinucleotide (P3)	10	AAC/GTT	118	0.71
		AAG/GTT	770	4.66
		AGC/CTG	1365	8.27
		AGG/CCT	617	3.74
		ATC/ATG	495	3.00
		Other	355	2.15
tetranucleotide (P4)	24	AAAG/CTTT	59	0.36
		ACAT/ATGT	37	0.22
		AGAT/ATCT	45	0.27
		AGCG/CGCT	94	0.57
		ATCG/ATCG	37	0.22
		Other	199	1.21
pentanucleotide (P5)	33	AAGAG/CTCTT	10	0.06
		ACGAG/CGTCT	26	0.16
		AGAGG/CCTCT	27	0.16
		Other	87	0.53
hexanucleotide (P6)	72	AAGCAG/CTGCTT	17	0.10
		ACACAT/ATGTGT	35	0.21
		AGAGGG/CCCTCT	12	0.07
		ATCGCC/ATGGCG	101	0.61
		Other	195	1.18
Total	141		16 511	100

**Figure 3.**
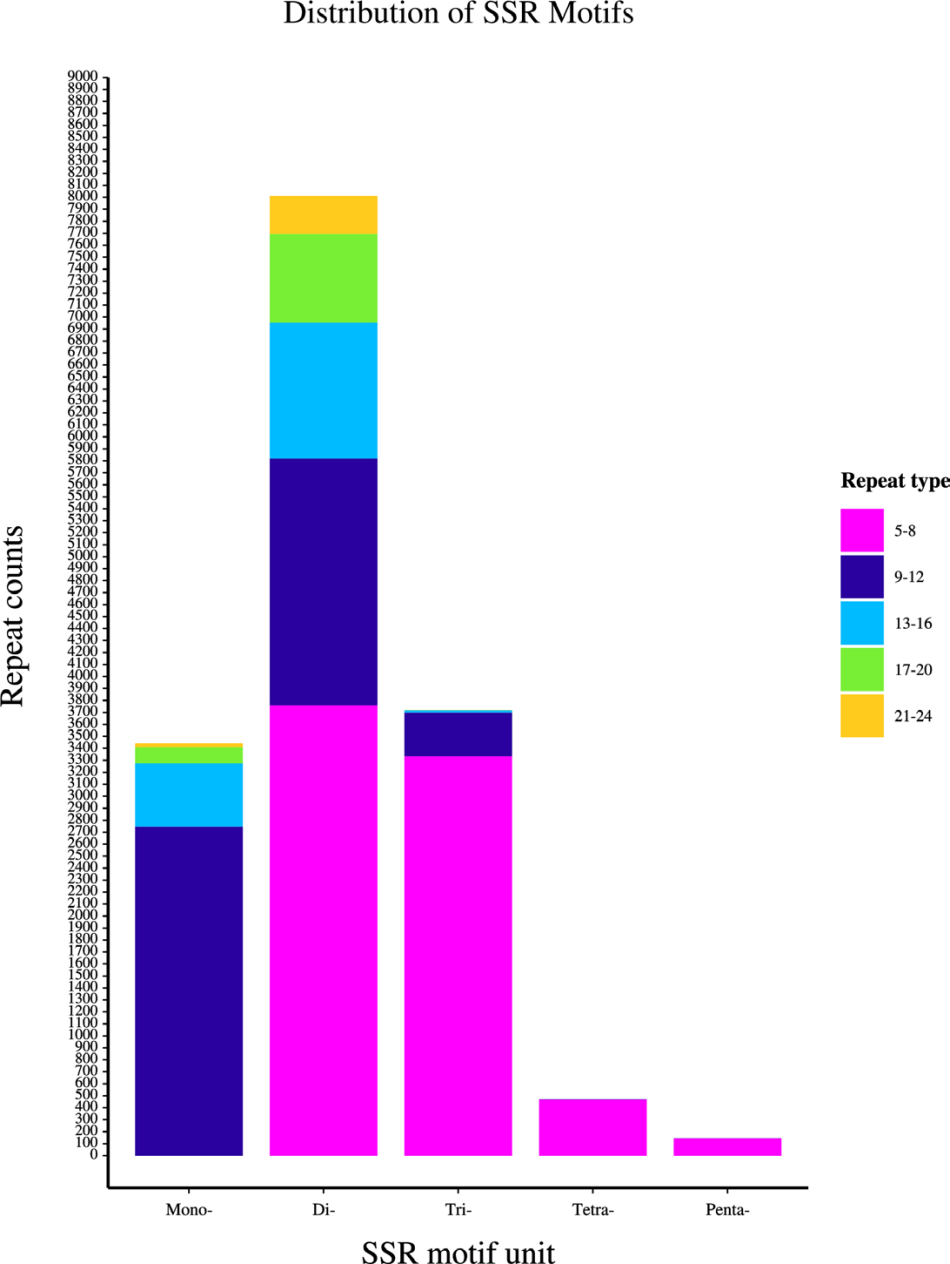
Distribution of repeat numbers across different SSR motif types in the transcriptome of *H. serrata*.

The distribution of SSR repeat counts was predominantly in the range of 5–20 repeats, which collectively accounted for 95.78% of all SSR loci. The 5–8 repeat class was the most abundant, representing 48.88% of total SSRs, and was predominantly composed of dinucleotide and trinucleotide motifs. The 9–12 repeat class was the second most common, comprising 31.41% of SSRs and consisting mainly of mononucleotide and dinucleotide repeats. Repeats in the 13–20 range were less frequent, accounting for only 15.49% of the total. SSRs with more than 20 repeats were the scarcest, representing a mere 4.21% of all loci (Fig. 3).

### Transcriptome SSR primers screening

**Figure 4.**
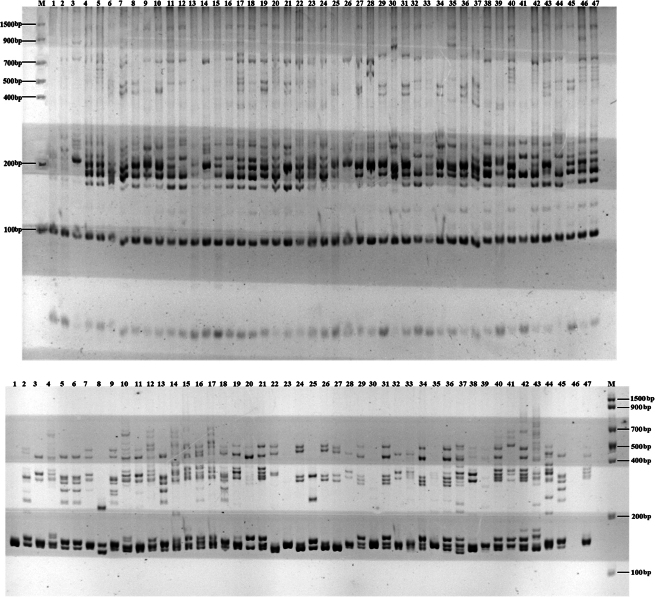
Partial electrophoretic map (primer Hup_09 and primer Hup_91). Marker is represented by M.

A set of 100 primer pairs was initially screened for polymorphism using DNA templates from *H. crispata* and *H. serrata*. The preliminary screening revealed that 65 pairs (65%) successfully amplified clear and reproducible bands. These 65 primers were subsequently used to amplify all 47 *Huperzia* DNA samples. The PCR products were separated by 8% non-denaturing polyacrylamide gel electrophoresis, which confirmed successful amplification for all primers. From these, 21 primers exhibiting clear polymorphism were selected for further analysis (Suppl. material 1: table SS2; Fig. 4), resulting in an effective primer rate of 32.30%.

### Analysis of polymorphism of SSR marker loci

The 21 highly polymorphic primers were used for genetic diversity analysis. Across the 47 *Huperzia* accessions, 98 polymorphic alleles were detected, averaging 4.7 alleles per locus. Nei’s gene diversity index (*H*) ranged from 0.1729 to 0.4906, and the average Shannon diversity index (*I*) was 0.4819. The polymorphism information content (PIC) of the 21 SSR loci varied from 0.3207 to 0.7877, with a mean value of 0.5891 (Table 3). Locus Hup_88 showed the minimum PIC value (0.3207), while Hup_28 showed the maximum (0.7877), indicating considerable variation in PIC values among the 21 polymorphic SSRs. These results demonstrate that the selected primers possess high polymorphism and that the sampled *Huperzia* accessions exhibit rich genetic diversity.

**Table 3.** Analysis of the SSR polymorphism in 21 pairs of the *Huperzia* genus.

Primer name	Allele number (*Na*)	Nei’s gene diversity (*H*)	Shannon diversity index (*I*)	Polymorphism information (*PIC*)
*Hup_09*	6	0.3764	0.5356	0.7385
*Hup_13*	5	0.3962	0.5819	0.6827
*Hup_14*	4	0.2748	0.4431	0.4975
*Hup_23*	6	0.2966	0.4551	0.7130
*Hup_27*	4	0.2537	0.4068	0.5157
*Hup_28*	7	0.4135	0.5999	0.7877
*Hup_33*	5	0.3086	0.4774	0.6778
*Hup_44*	4	0.2797	0.4483	0.5765
*Hup_45*	5	0.3166	0.4884	0.7057
*Hup_48*	5	0.3727	0.5547	0.6843
*Hup_53*	4	0.4906	0.6837	0.5914
*Hup_54*	4	0.4505	0.6414	0.5142
*Hup_56*	5	0.4843	0.6773	0.7003
*Hup_73*	6	0.2414	0.4001	0.6429
*Hup_76*	3	0.1729	0.3030	0.3864
*Hup_82*	4	0.2358	0.3747	0.5561
*Hup_87*	4	0.2540	0.3994	0.4456
*Hup_88*	5	0.2141	0.3539	0.3207
*Hup_90*	3	0.2212	0.3645	0.4306
*Hup_91*	6	0.2480	0.4008	0.6161
*Hup_93*	3	0.3572	0.5299	0.5884
Mean	4.7	0.3171	0.4819	0.5891

### Genetic structure analysis of *Huperzia*

**Figure 5.**
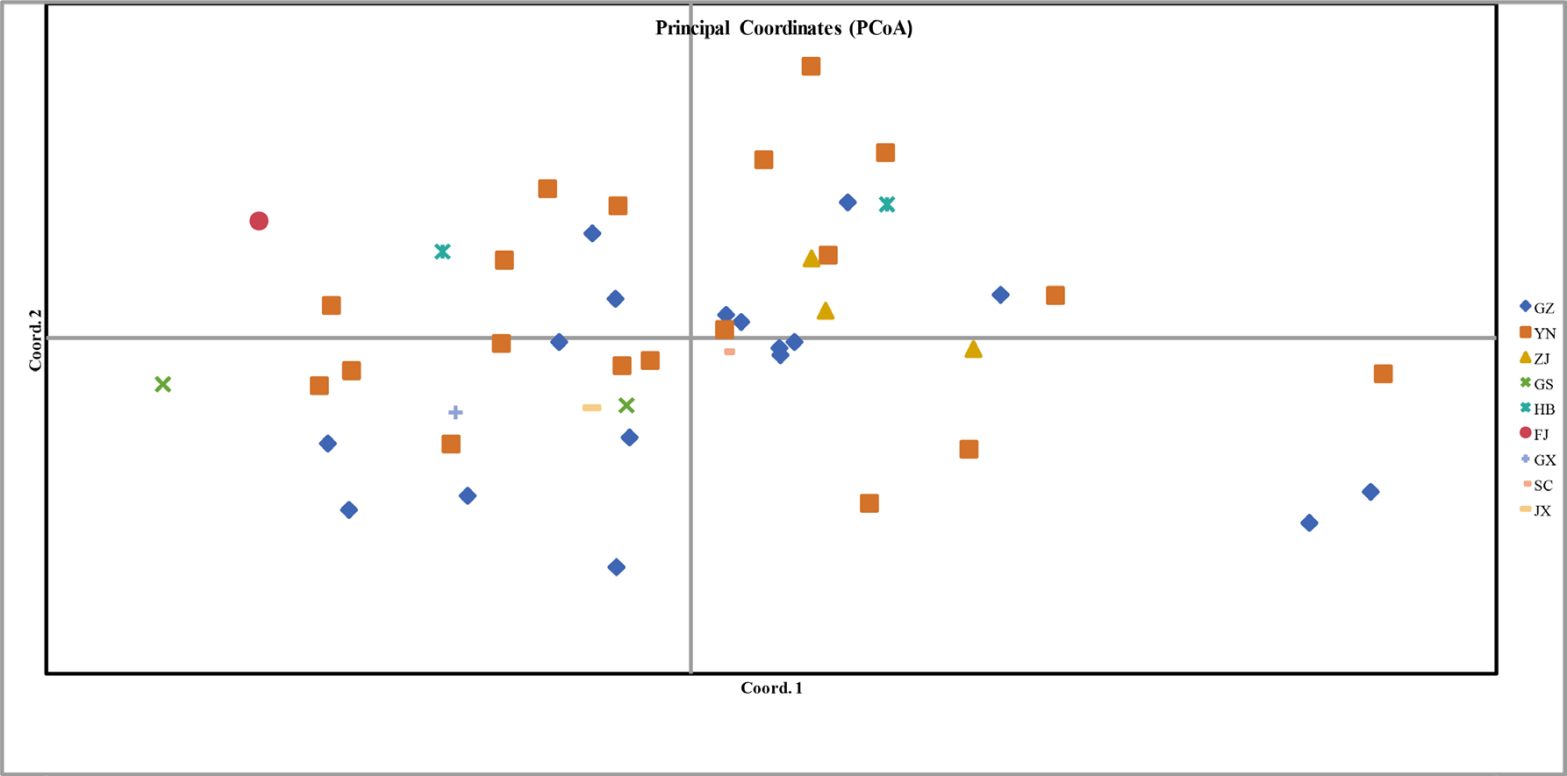
Principal coordinates analysis (PCoA) of 47 *Huperzia* accessions collected from fourteen regions in nine provinces of China. Note: A total of 47 *Huperzia* accessions were collected from fourteen different regions, with each region considered a distinct geographic sampling group in this study.

To assess genetic relationships, a principal coordinates analysis (PCoA) was conducted based on genetic distances among plants from nine different provinces using GenALEx (v6.503). In the resulting plot (Fig. 5), the spatial proximity between any two points reflects their genetic relatedness, with closer points indicating shorter genetic distances. However, regional sampling groups that were genetically close were not always in geographical proximity, suggesting a weak correlation between genetic and geographical distance in *Huperzia*. The banding patterns generated by the 21 polymorphic primers were scored in a binary (0/1) matrix using Excel. This matrix was used to calculate genetic similarity coefficients with the SIMILARITY module in NTSYS-pc (v2.10). The analysis revealed variation in genetic similarity among the different *Huperzia* regional sampling groups.

**Figure 6.**
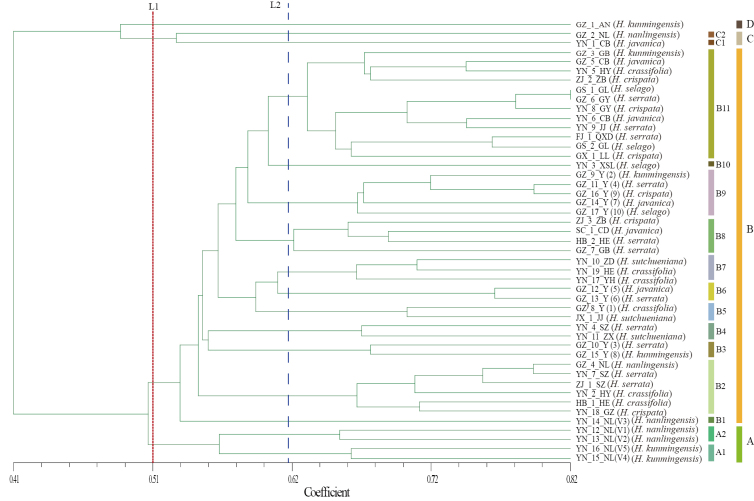
Dendrogram of 47 *Huperzia* accessions based on genetic similarity. Note: According to the cluster map, 47 germplasms were divided into four large groups, and the blue dotted line clearly divides them into 16 sections. Different groups have different colors.

The SSR markers exhibited a high degree of genetic polymorphism, yet their resolution at the species level displayed a complex pattern (Fig. 6). At a genetic similarity coefficient of 0.51 (Line L1), the tested samples were divided into four primary clusters (A–D). Cluster A demonstrated a relatively high degree of species aggregation, primarily comprising *H. nanlingensis* (YN_12 to YN_14) and *H. kunmingensis* (YN_15, YN_16) collected from Dali, Yunnan. This indicates that within specific geographic regions, SSR markers can effectively cluster closely related species with similar genetic backgrounds. Cluster B was the largest and most complex group, further subdivided into 11 sub-clusters (B1–B11). Individuals of *H. serrata* from Guizhou were clustered together with samples of the same species from Zhejiang or Fujian. Moreover, some *Huperzia serrata* and *H. javanica* from the same region are also clustered together. Clusters C and D contained a few individuals exhibiting unique genetic backgrounds; for instance, the *H. kunmingensis* sample from Anshun, Guizhou (GZ_1_AN), which fell into Cluster D, separated from the other samples at a similarity coefficient of 0.41. However, by further increasing the similarity coefficient threshold, the SSR markers could precisely distinguish groups of the same species from different geographic origins, demonstrating exceptionally high intra-specific and individual-level resolution.

## Discussion

### SSR distribution and sequence characteristics of the transcriptome of *H. serrata*

SSR markers serve as a powerful tool for authenticating species and assessing genetic diversity at the molecular level. By detecting variation in DNA repetitive sequences, they not only reveal genetic similarity and relatedness among species but also play a crucial role in the identification and conservation of species resources (Çilesiz 2025). These attributes establish SSR markers as a reliable and ideal technique, particularly for studying species with subtle or difficult-to-distinguish phenotypic characteristics (Preethi et al. 2020; Li et al. 2022). In this study, we conducted the first full-length transcriptome sequencing of *H. serrata*. From the 31,243 assembled unigenes, 16,511 SSR loci were identified, corresponding to an SSR frequency of 35.08%. This comprehensive dataset provides a valuable resource for future genetic studies and SSR marker development in *Huperzia*. The SSR frequency in *H. serrata* was found to be higher than that reported for other medicinal plants, such as the fern *Brainea insignis* (Blechnaceae, 20.58%) and the lycophyte *Lycopodiastrum casuarinoides* (Lycopodiaceae, 10.72%) (Liu et al. 2017; Chen et al. 2020). The variation in SSR frequency across plant species can be attributed to several factors, including species-specific genetic characteristics, differences in development methodologies and software, varying SSR search criteria, and the depth of DNA sequence resources (Wei et al. 2011). The high SSR frequency observed in the *H. serrata* transcriptome indicates a rich and diverse abundance of SSR loci, underscoring the utility of transcriptomic data for marker development in this genus.

The transcriptome of *H. serrata* was found to contain abundant microsatellites, encompassing mono- to hexanucleotide repeat motifs. Dinucleotide repeats were the predominant type, constituting 50.52% of all identified SSRs. This predominance of dinucleotide repeats is consistent with findings in other plant species, including the fern *Asplenium nidus* (Jia et al. 2016), as well as the medicinal plants *Viburnum japonicum* (Zhu et al. 2023), *Sambucus nigra* (Yao et al. 2019), *Lonicera japonica* (Jiang et al. 2012), and *Paeonia suffruticosa* (Luo et al. 2021). In contrast, the fern *Dicranopteris ampla* exhibits a distinct pattern, being primarily characterized by the AGG/CCT trinucleotide repeat motif (Liu et al. 2016).

The number of SSR repeats is generally positively correlated with locus polymorphism, whereby a higher repeat count often indicates greater potential for genetic variation (Chen et al. 2017). In this study, the vast majority (95.78%) of SSR repeats fell within the 5–20 range. Notably, repeats exceeding 16 units accounted for 9.51% of the total, suggesting a substantial number of SSRs with high repeat counts in the *H. serrata* transcriptome. This abundance of highly repetitive loci is highly advantageous for the development of polymorphic molecular markers and subsequent genetic analyses.

### Genetic diversity analysis of SSR loci

Genetic diversity forms the genetic foundation of species and serves as a critical resource for crop variety improvement, particularly for resources with a broad genetic base (Vilayheuang et al. 2016). In this study, the genetic diversity of 47 *Huperzia* accessions was evaluated using 21 polymorphic SSR primers. The number of alleles (Na) per locus ranged widely from 3 to 7, reflecting the degree of conservation at the SSR loci. According to the established criteria proposed by Botstein et al. (1980), markers with a polymorphism information content (PIC) greater than 0.5 are considered highly polymorphic, those between 0.25 and 0.5 are moderately polymorphic, and those below 0.25 exhibit low polymorphism. In our study, the PIC values ranged from 0.3207 to 0.7877, with a mean of 0.5891, indicating that the selected primers were highly polymorphic. The analysis also revealed a mean Nei’s gene diversity index (H) of 0.3171, and the Shannon’s information index (I) ranged from 0.3030 to 0.6837, with an average of 0.4819. Compared to the genetic diversity reported for other lycophytes, such as *Isoetes sinensis* (Chen and Wang 2006) and *Selaginella tamariscina* (Morajkar et al. 2022), the *Huperzia* germplasm examined in this study exhibited a relatively rich level of genetic diversity.

### Cluster analysis

Cluster analysis is a key tool in biodiversity and genetic research, effectively revealing genetic relationships and population structures within plant germplasm resources (Zhu et al. 2021). The findings from such analyses provide a scientific basis for the conservation, utilization, and breeding of these resources, thereby promoting the maintenance and sustainable use of biodiversity (Singh et al. 2025).

In multi-species phylogenetic investigations, clustering algorithms are theoretically expected to group accessions according to their evolutionary relationships and taxonomic identities, maintaining species monophyly across geographic ranges. However, in this study, the UPGMA cluster analysis based on SSR genetic similarity coefficients categorized the 47 morphologically authenticated *Huperzia* accessions into distinct groups that frequently violated strict taxonomic boundaries (e.g., *H. kunmingensis* and *H. nanlingensis*, both from Yunnan, form a cluster in the phylogenetic tree). This deviation from expected taxonomic monophyly can be attributed to three primary intersecting factors: the unique reproductive biology of lycophytes, the inherent taxonomic complexity of the *Huperzia* genus, and the profound limitations of SSR markers in deep phylogenetic analysis. In addition, there are reasons that cannot be ignored. Morphological classification is controversial, such as *H. serrata* and *H. javanica*, which were previously all classified as *H. serrata*. The same species has been isolated in different geographical environments for a long time, forming different varieties or subspecies, which leads to the misidentification of the accessions or taxonomic inconsistency.

First, *Huperzia* represents an ancient lineage of seedless vascular plants that rely entirely on spores for reproduction. Unlike seed plants, the microscopic spores of lycophytes are highly adapted for effective, long-distance wind dispersal (Huang and He 2010). This reproductive strategy facilitates extensive gene flow across geographically proximate regions (Ebihara and Nitta 2019). Consequently, sympatric populations of different species may experience frequent genetic exchange or share a recent, localized ancestral gene pool. This spore-driven regional gene flow causes geographic genetic signals to occasionally overpower deeper phylogenetic boundaries, resulting in localized genetic similarities among different species within the same geographic niche.

Second, the morphological identification and delimitation of *Huperzia* species, particularly within the *H. serrata* complex, is notoriously challenging and fraught with taxonomic uncertainty. The genus is characterized by extremely high phenotypic plasticity in response to micro-environmental conditions, alongside a complex history of frequent hybridization and reticulate evolution (Shrestha and Zhang 2015). Taxonomic boundaries have long been debated, as many morphological variations and cryptic species exist within what was traditionally considered a single biological unit. This high morphological and genetic complexity means that some degree of taxonomic ambiguity is virtually unavoidable during initial specimen authentication, which directly contributes to the polyphyletic appearance of the clustering dendrogram.

Finally, SSR molecular markers inherently possess some methodological limitations when applied across species boundaries (Tran et al. 2020). While SSRs are exceptionally polymorphic and powerful for fine-scale population genetics and tracking intraspecific gene flow, their hypermutability inevitably leads to allele size homoplasy over extended evolutionary timescales. More critically, the genus *Huperzia* is heavily impacted by frequent polyploidization events (Guo et al. 2024). Standard SSR distance matrices are mathematically unequipped to handle variable ploidy levels among taxa. The presence of duplicated genomes across accessions violates the assumptions of standard diploid clustering algorithms, forcing mathematically skewed and phylogenetically inaccurate groupings.

In conclusion, while SSR markers demonstrate excellent resolution at the individual and intra-specific levels, a single panel of nuclear microsatellites is fundamentally insufficient to resolve the deep evolutionary relationships of the *Huperzia* genus. To accurately delimit species boundaries and overcome the confounding variables of spore-mediated gene flow, morphological ambiguity, and nuclear polyploidy, future systematic studies must transition toward the utilization of relatively conserved, maternally inherited chloroplast genome (cpDNA) sequences (e.g., *rbcL*, *matK*, and *psbA–trnH*) alongside rigorous morphological revisions.

## Conclusion

This study presents the first comprehensive transcriptomic analysis of the species *H. serrata* using PacBio SMRT sequencing, leading to the successful development of a set of novel SSR markers. The identification of 16,511 SSR loci underscores the abundance of microsatellites within the *H. serrata* transcriptome. Crucially, the subsequent application of these newly developed markers to 47 accessions, representing diverse species and regional sampling groups within the genus *Huperzia*—demonstrated their high cross-species transferability. This broad applicability effectively revealed the interspecific phylogenetic relationships and intraspecific genetic diversity within the genus. The successful cross-amplification of these SSRs highlights their significant value for plant systematics. Our findings demonstrate that SSR cluster analysis is a highly effective tool for assessing genetic diversity and classifying germplasm resources at both the species and population levels. However, there are some inconsistencies between cluster analysis and morphological classification and identification, so it seems that this method (SSR) cannot be used to identify different species.

The molecular markers developed herein provide a powerful means to conduct germplasm identification, genetic diversity analysis, and DNA fingerprinting. This work establishes a critical foundation for formulating informed breeding and conservation strategies for *Huperzia* species, thereby contributing to the broader goal of biodiversity conservation and sustainable utilization.

As a preliminary investigation into SSR markers derived from *H. serrata* for broader application in its allied taxa, this study paves the way for future systematic and evolutionary research. Subsequent efforts should focus on developing a wider array of polymorphic primers and incorporating a more comprehensive taxonomic sampling across the genus *Huperzia* to further validate and expand upon these findings in species delimitation and phylogenetic reconstruction.

## Additional information

### Artificial Intelligence (AI) use

The authors accept full responsibility for the content of the manuscript, including the disclosure of any use of AI.

No AI tools were used in the preparation of this manuscript.

### Author ORCIDs

D. Wang https://orcid.org/0009-0008-5997-7787

### Data availability

Transcriptome sequencing data related to *H. serrata* have been submitted to the NGDC, BioProject number: PRJCA022941, accessible at https://ngdc.cncb.ac.cn/search/specific?db=bioproject&q=PRJCA022941/.

## Supplementary materials

Supplementary material 1 Information of *Huperzia* samples from different regions and SSR marker information of 21 primers Data type docx https://binary.pensoft.net/file/1607163This dataset is made available under the Open Database License (http://opendatacommons.org/licenses/odbl/1.0/). The Open Database License (ODbL) is a license agreement intended to allow users to freely share, modify, and use this Dataset while maintaining this same freedom for others, provided that the original source and author(s) are credited. Muzi Li, Dengpan Yin, Haibo Li, Yumo Yue, Haibin Zhou, Wei Yu, Rui Qin, Zhongnian Zhang, Jiahao Wen, Yeqin Xu, Chaokai Miao, Qinyuan Shen, Dekai Wang
