# Supplementary material for: Evaluation of genetic diversity and structure of the endangered medicinal plant genus Huperzia (Lycopodiaceae) in China based on EST–SSR markers
Source: PhytoKeys. 2026 Apr 24;274:31–48. doi: 10.3897/phytokeys.274.180449 (PMC13135199; doi:10.3897/phytokeys.274.180449)
Supplement: Supplementary material 1 — Information of Huperzia samples from different regions and SSR marker information of 21 primers [file phytokeys-274-031_article-180449__-s001.docx]

**Table S1 Information of *Huperzia* samples from different regions.**

| **Number** | **Source** | **Sampling date** | **Longitude** | **Latitude** | **Species** |
| --- | --- | --- | --- | --- | --- |
| GZ_1_AN | Anshun, Guizhou | 2021.10.15 | 105.552°E‌ | 26.145°N | *Huperzia kunmingensis* |
| GZ_2_NL | Liupanshui, Guizhou | 2023.09.20 | 104.83°E‌ | *26.592*°N | *Huperzia nanlingensis* |
| YN_1_CB | Shaotong, Yunnan | 2023.10.21 | 103.717°E‌ | 27.338°N | *Huperzia javanica* |
| GZ_3_GB | Guiyang, Guizhou | 2021.10.15 | 106.42°E‌ | 26.357°N | *Huperzia kunmingensis* |
| GZ_4_NL | Anshun, Guizhou | 2023.04.24 | 105.552°E‌ | 26.145°N | *Huperzia nanlingensis* |
| YN_2_HY | Dali, Yunnan | 2023.04.24 | 100.241°E‌ | *25.593*°N | *Huperzia crassifolia* |
| YN_3_XSL | Dali, Yunnan | 2023.04.24 | 100.241°E‌ | *25.593*°N | *Huperzia selago* |
| YN_4_SZ | Shaotong, Yunnan | 2021.10.15 | 103.717°E‌ | 27.338°N | *Huperzia serrata* |
| HB_1_HE | Enshi, Hubei | 2022.04.13 | 109.479°E‌ | *30.295*°N | *Huperzia crassifolia* |
| GZ_5_CB | Liupanshui, Guizhou | 2023.09.20 | 104.83°E‌ | *26.592*°N | *Huperzia javanica* |
| GZ_6_GY | Liupanshui, Guizhou | 2023.09.20 | 104.83°E‌ | *26.592*°N | *Huperzia serrata* |
| YN_5_HY | Shaotong, Yunnan | 2023.10.21 | 103.717°E‌ | 27.338°N | *Huperzia crassifolia* |
| ZJ_1_SZ | Yuyao, Zhejiang | 2022.09.12 | 121.154°E‌ | *30.037*°N | *Huperzia serrata* |
| GS_1_GL | Longnan, Gansu | 2021.09.22 | 104.926°E‌ | *33.392*°N | *Huperzia selago* |
| YN_6_CB | Shaotong, Yunnan | 2023.10.21 | 103.717°E‌ | 27.338°N | *Huperzia javanica* |
| YN_7_SZ | Dali, Yunnan | 2023.04.24 | 100.241°E‌ | *25.593*°N | *Huperzia serrata* |
| YN_8_GY | Dali, Yunnan | 2023.04.24 | 100.241°E‌ | *25.593*°N | *Huperzia crispata* |
| GZ_7_GB | Liupanshui, Guizhou | 2023.09.20 | 104.83°E‌ | *26.592*°N | *Huperzia serrata* |
| YN_9_JJ | Qujing, Yunnan | 2021.10.15 | 103.796°E‌ | 25.489°N | *Huperzia serrata* |
| ZJ_2_ZB | Yuyao, Zhejiang | 2022.09.12 | 121.154°E‌ | *30.037*°N | *Huperzia crispata* |
| YN_10_ZD | Dali, Yunnan | 2023.04.24 | 100.241°E‌ | *25.593*°N | *Huperzia sutchueniana* |
| YN_11_ZX | Dali, Yunnan | 2023.04.24 | 100.241°E‌ | *25.593*°N | *Huperzia sutchueniana* |
| YN_12_NL(V1) | Dali, Yunnan | 2023.04.24 | 100.241°E‌ | *25.593*°N | *Huperzia nanlingensis* |
| YN_13_NL(V2) | Dali, Yunnan | 2023.04.24 | 100.241°E‌ | *25.593*°N | *Huperzia nanlingensis* |
| YN_14_NL(V3) | Dali, Yunnan | 2023.04.24 | 100.241°E‌ | *25.593*°N | *Huperzia nanlingensis* |
| YN_15_NL(V4) | Dali, Yunnan | 2023.04.24 | 100.241°E‌ | *25.593*°N | *Huperzia kunmingensis* |
| YN_16_NL(V5) | Dali, Yunnan | 2023.040.24 | 100.241°E‌ | *25.593*°N | *Huperzia kunmingensis* |
| GZ_8_Y (1) | Liupanshui, Guizhou | 2021.10.15 | 104.83°E‌ | *26.592*°N | *Huperzia crassifolia* |
| GZ_9_Y (2) | Liupanshui, Guizhou | 2021.10.15 | 104.83°E‌ | *26.592*°N | *Huperzia kunmingensis* |
| GZ_10_Y (3) | Liupanshui, Guizhou | 2021.10.15 | 104.83°E‌ | *26.592*°N | *Huperzia serrata* |
| GZ_11_Y (4) | Liupanshui, Guizhou | 2021.10.15 | 104.83°E‌ | *26.592*°N | *Huperzia serrata* |
| GZ_12_Y (5) | Liupanshui, Guizhou | 2021.10.15 | 104.83°E‌ | *26.592*°N | *Huperzia javanica* |
| GZ_13_Y (6) | Liupanshui, Guizhou | 2021.10.15 | 104.83°E‌ | *26.592*°N | *Huperzia serrata* |
| GZ_14_Y (7) | Liupanshui, Guizhou | 2021.10.15 | 104.83°E‌ | *26.592*°N | *Huperzia javanica* |
| GZ_15_Y (8) | Liupanshui, Guizhou | 2021.10.15 | 104.83°E‌ | *26.592*°N | *Huperzia kunmingensis* |
| GZ_16_Y (9) | Liupanshui, Guizhou | 2021.10.15 | 104.83°E‌ | *26.592*°N | *Huperzia crispata* |
| GZ_17_Y (10) | Liupanshui, Guizhou | 2021.10.15 | 104.83°E‌ | *26.592*°N | *Huperzia selago* |
| YN_17_YH | Honghe, Yunnan | 2021.10.15 | 103.374°E‌ | *23.363*°N | *Huperzia crassifolia* |
| ZJ_3_ZB | Yuyao, Zhejiang | 2022.09.12 | 121.154°E‌ | *30.037*°N | *Huperzia crispata* |
| FJ_1_QXD | Sanming, Fujian | 2021.12.08 | 116.226°E‌ | *25.305*°N | *Huperzia serrata* |
| GX_1_LL | Longlin, Guangxi | 2021.12.22 | 105.344°E‌ | *24.770*°N | *Huperzia crispata* |
| GS_2_GL | Longnan, Gansu | 2021.09.22 | 104.926°E‌ | *33.392*°N | *Huperzia selago* |
| SC_1_CD | Chengdu, Sichuan | 2021.12.20 | 104.066°E‌ | 30.573°N | *Huperzia javanica* |
| JX_1_JJ | Jiujiang, Jiangxi | 2021.12.04 | 115.992°E‌ | 29.712°N | *Huperzia sutchueniana* |
| YN_18_GZ | Shaotong, Yunnan | 2021.10.15 | 103.717°E‌ | 27.338°N | *Huperzia crispata* |
| HB_2_SZ | Enshi, Hubei | 2022.04.13 | 109.479°E‌ | *30.295*°N | *Huperzia serrata* |
| YN_19_HE | Shaotong, Yunnan | 2021.10.15 | 103.717°E‌ | 27.338°N | *Huperzia crassifolia* |

**Table S2 SSR marker information of 21 primers.**

| **Primer name** | **motif** | **Primer sequence** | **Product length/bp** |
| --- | --- | --- | --- |
| *Hup_09* | (T)18 | F:GATTGTAGGCCGCCTTCTTA  R:TGATGGTGATCTCGGATTGA | 249 |
| *Hup_13* | (GA)39 | F:AAAGGTAAACTCGGATGGGC  R:ACTTGGAGCAGCCACAGATT | 238 |
| *Hup_14* | (A)27 | F:GATATTTGTGTAGGGCCGGA  R:CCAAATTCTGACGTGCAATG | 164 |
| *Hup_23* | (T)18 | F:AAAGCAAAGTTTTTGCGCC  R:TAGGAGGGGTAACGGAGGTT | 254 |
| *Hup_27* | (TA)23 | F:ACCCATGCGAGCATTACATT  R:GAAAAATTCCGTACCGTTGG | 184 |
| *Hup_28* | (TA)29 | F:AGCCAAGAGCTTGGTGAGAG  R:AATGTTCACGCAAAAGGAGG | 237 |
| *Hup_33* | (TC)68 | F:CCCTAGGGTATGAAAAAGGCA  R:TGGAGGCATAAGGAGTGTGA | 270 |
| *Hup_44* | (GGA)8 | F:AGGAGGGCATGGAGAGTTTC  R:GAAGGCGGAACAAGAGACAG | 173 |
| *Hup_45* | (AG)30 | F:GTAGATGTCAGCCGGGTAGG  R:ACCAGAAACCCTTGCTCTCC | 274 |
| *Hup_48* | (TA)27 | F:AAGGGCAGAACGACATAACG  R:CGATTGGGTCTCCTTCAGAC | 188 |
| *Hup_53* | (A)16 | F:GGTATTTTGGGGTAGCAGCA  R:GGAAATTGGCAGGTATGCTC | 258 |
| *Hup_54* | (TC)39(AC)7gca(CG)6 | F:GCAACGAGTTTGCAAAACAA  R:GGCCAAAAGCAAGAAAACAT | 278 |
| *Hup_56* | (CA)61 | F:CCTTCGGGTATTTAATAGACGTT  R:TTGGATTAGTTATGAACGAGGC | 244 |
| *Hup_73* | (T)18 | F:AAAGCAAAGTTTTTGCGCC  R:TAGGAGGGGTAACGGAGGTT | 254 |
| *Hup_76* | (TC)18 | F:TTGCTTTTGCTGGGATTTTT  R:CTTTGACAGCTGACGTGCAT | 232 |
| *Hup_82* | (GAAGAC)8 | F:AAAGGTGGAAGTTGTCACGG  R:CGTCTTCATCATCAGCCTCA | 170 |
| *Hup_87* | (ATCACC)7 | F:GCTGACGGTCTTCGTCTTCT  R:ATCTCCTTCGCCTCAGACAA | 210 |
| *Hup_88* | (CAGCAA)9 | F:AGGCAAAAGGCTATGCAAGA  R:ACATTCTCCGCATTTTGCTC | 188 |
